# Auditing chronic disease care: Does it make a difference?

**DOI:** 10.4102/phcfm.v7i1.753

**Published:** 2015-06-26

**Authors:** Vivien Essel, Unita van Vuuren, Angela De Sa, Srini Govender, Katie Murie, Arina Schlemmer, Colette Gunst, Mosedi Namane, Andrew Boulle, Elma de Vries

**Affiliations:** 1 Public Health Registrar, University of Cape Town, South Africa; 2 Western Cape Provincial Health Services, South Africa; 3 Chronic Disease Management, Western Cape Provincial Health Services, South Africa; 4 Family Physician, University of Cape Town, South Africa; 5 Western Cape Metro District Health Services, South Africa; 6 Cape Winelands District Health Services, Western Cape Government: Health, South Africa; 7 Family Physician, Stellenbosch University, South Africa; 8 Public Health Specialist, University of Cape Town, South Africa

## Abstract

**Background:**

An integrated audit tool was developed for five chronic diseases, namely diabetes, hypertension, asthma, chronic obstructive pulmonary disease and epilepsy. Annual audits have been done in the Western Cape Metro district since 2009. The year 2012 was the first year that all six districts in South Africa's Western Cape Province participated in the audit process.

**Aim:**

To determine whether clinical audits improve chronic disease care in health districts over time.

**Setting:**

Western Cape Province, South Africa.

**Methods:**

Internal audits were conducted of primary healthcare facility processes and equipment availability as well as a folder review of 10 folders per chronic condition per facility. Random systematic sampling was used to select the 10 folders for the folder review. Combined data for all facilities gave a provincial overview and allowed for comparison between districts. Analysis was done comparing districts that have been participating in the audit process from 2009 to 2010 (‘2012 old’) to districts that started auditing recently (‘2012 new’).

**Results:**

The number of facilities audited has steadily increased from 29 in 2009 to 129 in 2012. Improvements between different years have been modest, and the overall provincial average seemed worse in 2012 compared to 2011. However, there was an improvement in the ‘2012 old’ districts compared to the ‘2012 new’ districts for both the facility audit and the folder review, including for eight clinical indicators, with ‘2012 new’ districts being less likely to record clinical processes (OR 0.25, 95% CI 0.21–0.31).

**Conclusion:**

These findings are an indication of the value of audits to improve care processes over the long term. It is hoped that this improvement will lead to improved patient outcomes.

## Abstrait

**Vérification des soins pour maladies chroniques: Cela fait-il une différence?**

**Contexte:**

Un instrument de vérification intégré a été conçu pour cinq maladies chroniques, telles que le diabète, l’hypertension, l’asthme, les maladies pulmonaires obstructives chroniques et l’épilepsie. Des vérifications annuelles ont été effectuées depuis 2009 dans la région métropolitaine du Western Cape. C’est en 2012 que pour la première fois les six districts de la Province du Western Cape en Afrique du Sud ont pris part au processus de vérification.

**Objectif:**

Déterminer si les vérifications cliniques améliorent les soins pour maladies chroniques dans les districts sanitaires avec le temps.

**Lieu:**

Province du Western Cape, Afrique du Sud.

**Méthodes:**

On a fait des vérifications internes des processus et de la disponibilité des équipements dans les établissements de soins primaires, ainsi qu’une vérification de 10 dossiers par condition chronique par établissement. On a utilisé un programme d’échantillonnage aléatoire systématique pour sélectionner les 10 dossiers à vérifier. Les données combinées de tous les établissements ont fourni un aperçu provincial et permis de comparer les districts. On a fait une analyse pour comparer les districts qui ont pris part au processus de vérification de 2009 à 2010 (‘2012 anciens’) aux districts qui ont commencé la vérification récemment (‘2012 nouveaux’).

**Résultats:**

Le nombre d’établissements vérifiés a augmenté progressivement de 29 en 2009 à 129 en 2012. Les améliorations entre les différentes années sont modestes et la moyenne générale provinciale semble pire en 2012 qu’en 2011. Cependant, on remarque une amélioration dans les districts “2012 anciens” par rapport aux districts ‘2012 nouveaux’ pour la vérification des établissements et la vérification des dossiers, y compris huit indicateurs cliniques, avec les districts ‘2012 nouveaux’ qui sont moins susceptibles d’enregistrer les processus cliniques (OR 0.25, 95% CI 0.21–0.31).

**Conclusion:**

Ces résultats sont une indication de la valeur des vérifications pour améliorer les processus à long terme. Nous espérons que cette amélioration se traduira par de meilleurs résultats chez les patients.

## Introduction

Globally there is a rapidly increasing burden of non-communicable diseases (NCDs). It is expected that by the year 2020, NCDs will account for 57% of the global burden of disease. This will be an increase of 11% from the 2001 figure of 46%.^1^ Previously seen as diseases of the wealthy, NCDs are now a significant problem amongst the world's poor, especially in sub-Saharan Africa (SSA).

The age-standardised mortality rate due to NCDs was found to be higher in four SSA countries (Democratic Republic of Congo, Nigeria, Ethiopia and South Africa) compared to wealthier countries with a higher income.^2^ South Africa at the moment is characterised by a quadruple burden of communicable, non-communicable, perinatal and maternal diseases, and injury-related disorders with NCDs, specifically cardiovascular diseases, type 2 diabetes, cancer, chronic lung disease and depression are on the rise in both rural and urban settings.^3^ In South Africa's Western Cape Province from 2003 to 2006 pooled estimates of causes of death found NCDs to be the main cause of death amongst adults aged 40 years and older.^4^

There is increasing demand for chronic care at health facilities, and this is putting strain on services. Measures are needed to address the growing burden of NCDs in South Africa and in the Western Cape Province; without them it is estimated that NCDs will rise considerably in South Africa over the next decades.^3^

One such measure is the use of clinical audits as part of a surveillance system to improve the quality of care given to patients, especially in primary healthcare (PHC) settings.^5,6^ The purpose of a clinical audit is to evaluate and measure one's own practice against a recognised professional standard. It reminds clinicians of the available standards and guidelines that relate to their practice, and identifies training needs.^7,8^ Using clinical audits to improve services is not a new concept. Several studies including a meta-analysis have shown significant improvements in health services that were audited compared to those that were not.^9,10,11^ Where there were improvements, the effects seen were generally small to moderate.^8,12^ An evaluation of diabetic audits done from 2005 to 2009 in Cape Town, Western Cape, South Africa found an improvement in all nine clinical processes. The findings from this study showed that in resource-limited areas quality improvement can be attained by doing clinical audits.^13^

In the Western Cape Province NCDs account for a higher proportion of deaths in adults (58%) than seen nationally in the country (38%).^14^ This prompted the Western Cape Department of Health to consider the management of people with NCDs comprehensively. For this reason the annual integrated chronic disease audit was established for five chronic diseases, namely diabetes, hypertension, asthma, chronic obstructive pulmonary disease (COPD) and epilepsy. The Integrated Audit for Chronic Disease Management follows from the work done on the Cardiovascular Risk Factor or Diabetic Audit that has been done in its Metro District since 2005.^13,15^

As part of a quality improvement project, clinical governance structures in the department proposed that an integrated audit be done. An annual integrated chronic disease audit which looks at chronic disease care and management of risk factors enables the Department of Health to identify gaps and strengthen its health systems, specifically PHC. Currently a separate HIV and/or AIDS, sexually transmitted diseases (STIs) and tuberculosis (TB) (HAST) audit is done annually in the province. In future it is envisioned for the Western Cape Province to have a truly integrated audit which will include all chronic conditions such as mental health and HIV infection.

The aim of this study was to assess the effectiveness of clinical audits done for NCD care over time in the Western Cape Province by comparing districts that have being participating in the audit process from 2009 and 2010 compared to districts that started auditing recently (2011 and/or 2012).

## Methods

### Study design

This cross-sectional study was an internal audit where facility staff members audited themselves. It is believed that by involving them, they will take ownership of the process and use the results to improve services at their own facilities. Staff members included family physicians, senior medical officers or clinical nurse practitioners at each facility. Family physicians, who were the facilitators for the audit process, held an annual teaching seminar prior to the audit where every staff member who was involved in the audit was trained in a standardised manner on the appropriate collection of data.

### Setting and sampling

In the Western Cape Province there are six health districts (the Metro, Cape Winelands, Eden, Central Karoo, West Coast and Overberg). Chronic care in these six districts is mainly provided at PHC facilities. In 2012 there were 326 PHC facilities providing services to patients with NCDs. All six districts participated in the audit in 2012 and each district was asked to list the facilities providing chronic care in their district that would participate in the audit. Selection of facilities was based on feasibility to perform the audit.

Two components of the audit were evaluated using a standardised chronic disease audit tool. The two components looked at the facility's process and equipment availability as well as a folder review of five chronic conditions. Facility process and equipment involved auditing patient preparation rooms, consulting rooms, clinical management processes and access to equipment used in chronic care. For the folder review, in each facility random systematic sampling was used to select 10 folders per chronic condition (diabetes, hypertension, asthma, COPD and epilepsy). It was a pragmatic decision to select 10 folders per chronic condition per facility. This number was considered feasible for facilities and also sufficient to monitor trends in care processes across facilities within a given sub-district. Folders were eligible for selection if the adult patient had been receiving treatment at the PHC facility for at least one year. Folder review per chronic condition looked at a set of fundamental chronic care indicators based on national guidelines for the different conditions, the Standard Treatment guidelines and Essential Medicines List for South Africa^16,17,18,19,20^ as well as the criteria set in the Primary Care 101 guidelines, a symptom-based approach to the adult in PHC.^21^

Data were collected in February of each year of 2009 to 2012.

### Data analysis

Data were analysed using the statistical programme Stata version 12.1 (StataCorp, College Station, Texas). Data collected were in a binary format where a positive response was given to the presence of specified indicators. With the unit of analysis being the facility, the final score for each district was obtained from the average score for all participating facilities within that district. Pooled district scores gave rise to provincial totals for each year.

Initial exploratory analysis showed that the data were not normally distributed and hence median percentages were used to present the results of facility audit processes and folder review. To determine the effectiveness of audits results for the years from 2009 to 2012 were compared, and for 2012 pooled results for districts that have been auditing since 2009 and 2010 (Metro, Eden and Cape Winelands), referred to as ‘2012 old’, were compared to districts that started auditing recently in 2011 and 2012 (West Coast, Overberg and Central Karoo), referred to as ‘2012 new’. Descriptive statistics with inter-quartile ranges and bar graphs were used to show the changes in the audit results over the years. To test the statistical significance between ‘2012 old’ and ‘2012 new’ results the Mann-Whitney non-parametric test was used.^22^

For the 2012 audit results data were expanded from percentage scores per question to binary responses by folder, enabling a logistic regression model to be fitted with history of previous audits (new versus old); rural (Eden, Cape Winelands, West Coast, Overberg and Central Karoo) versus the Metro district and comparing selected indicators for the five chronic diseases (HbA_1C_ and foot exam for diabetes; serum creatinine and random total cholesterol for hypertension; control of asthma for asthma; counselling for smokers and counselling on inhaler use for COPD; and number of visits recording seizures for epilepsy). Data were clustered on patient folders to account for where responses to more than one question were from the same folder, ensuring robust standard errors.

### Ethical considerations

Ethics approval for the annual audit was granted by the University of Cape Town's Research Ethics Committee in 2009 (HREC Ref.: 181/2009). In order to continue auditing and to publish this evaluation annual extensions have been given.

## Results

The number of facilities participating in the audit has increased from 2009 to 2012 and is represented in Table 1.

**TABLE 1:** Participating facilities per district, 2009–2012.

Year	Metro	Eden	Cape Winelands	Overberg	West Coast	Central Karoo	Total
2009	29	0	0	0	0	0	**29**
2010	33	2	3	0	0	0	**38**
2011	43	7	11	0	5	12	**78**
2012	46	12	14	24	24	9	**129**

**FIGURE 1:**
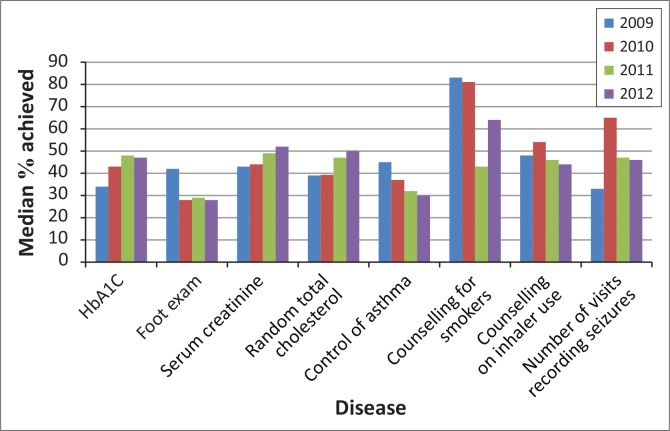
Folder audit per chronic disease, 2009–2012.

**FIGURE 2:**
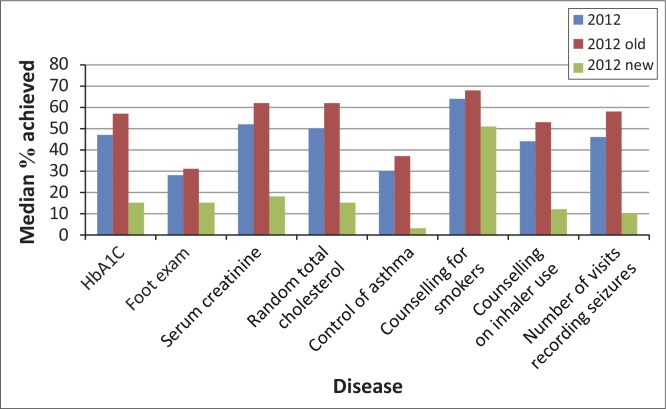
Folder audit 2012, comparing ‘2012 old’ and ‘2012 new’ districts.

Improvements in the audit process from 2009 to 2012 were minimal (Table 2 and Figure 1). The overall results may not show evidence of improvement when comparing 2011 to 2012, but if a comparison is made of ‘2012 old’ districts with ‘2012 new’ districts, improvements can be seen (Table 2 and Figure 2). With regard to the facility audit presented in Table 2, the patient preparation room was well stocked.

**TABLE 2:** Facility audit, 2009–2012.

Variable			Median percentage achieved per process			
**Year (Number of participating facilities)**	2009 (29)	2010 (38)	2011 (78)	2012 (129)	‘2012 old’ (72)	‘2012 new’ (57)
**Consulting rooms**						
Standard BP cuff	88	84	87	92	93	91
Cuff for the obese	49	45	50	45	47	39
Footscreening forms	68	58	73	63	71	38
Peak expiratory flow meter	60	53	59	56	58	51
**Patient preparation rooms**						
Functioning scale	100	100	96	99	99	100
Height chart	100	90	91	99	100	96
BMI chart or wheel	69	66	74	84	81	93
Urine dipsticks	97	100	98	100	100	99
Glucometer	97	100	96	100	100	100
**Access to equipment**						
Monofilaments for foot exam	90	73	74	78	84	57
Snellen Chart (normal)	97	100	96	94	95	93
ECG machine	100	96	89	76	88	39
**Processes**						
Chronic Disease Register	83	83	89	77	84	54
Chronic care team	61	73	63	50	57	25
Group health education	70	90	78	80	84	66
Community support groups	62	70	76	74	80	54

BP, blood pressure; BMI, body mass index; ECG, electrocardiogram.

In the consulting rooms, the availability of blood pressure cuffs for the obese was poor. In 2012 only 45% of audited consulting rooms had cuffs for the obese, with the ‘2012 old’ districts achieving 47% and ‘2012 new’ districts only achieving 39%. The 2012 provincial average achieved 50% of facilities having chronic care teams. However, this was mainly due to what was achieved in the ‘2012 old’ districts (57%) rather than in the ‘2012 new’ districts (25%).

Figure 1 shows the median proportions achieved for certain indicators per chronic disease for the folder review done from 2009 to 2012. Generally proportions achieved for the overall provincial average in 2012 in most of the chronic disease indicators (except serum creatinine and counselling for smokers) were less than 50%. However, higher proportions were achieved in the ‘2012 old’ districts compared to the ‘2012 new’ districts (Table 3 and Figure 2).

**TABLE 3:** Folder audit comparing ‘2012 old’ and ‘2012 new’ districts.

Variables	Median percentage achieved (IQR)	2012 total	2012 old	2012 new	Mann-Whitney test/ *p*-value
Number					
Number of folders audited	-	6450	3600	2850	-
Number of facilities	-	129	72	57	-
Diabetes					
HbA_1C_	47	-	57 (50–63)	15 (7–24)	< 0.001
Foot exam	28	-	31 (19–37)	15 (2–30)	0.029
Hypertension					
Serum creatinine	52	-	62 (55–70)	18 (8–25)	< b0.001
Random total cholesterol	50	-	62 (50–68)	15 (8–23)	< 0.001
Asthma					
Control of asthma	30	-	37 (27–45)	3 (0–4)	< 0.001
COPD					
Counselling for smokers	64	-	68 (64–80)	51 (38–68)	0.016
Counselling on inhaler use	44	-	53 (31–70)	12 (0–20)	< 0.001
Epilepsy					
Number of visits that recorded seizures	46	-	58 (48–63)	10 (3–14)	< 0.001

IQR, interquartile range.

Although the provincial average for 2012 showed that HbA_1C_ and foot exam in diabetics were done at poor rates (47% and 28% respectively), ‘2012 old’ districts achieved more than the ‘2012 new’ districts (Table 3). For hypertensive care the number of folders that recorded serum creatinine having been measured was 62% in the ‘2012 old’ districts and only 18% in the ‘2012 new’, with that of random total cholesterol being 62% and 15% in ‘2012 old’ and ‘2012 new’ respectively. Improvements were also seen when ‘2012 old’ districts were compared to ‘2012’ new districts for the folder review of asthma, COPD and epilepsy.

Across all five chronic disease indicators ‘2012 new’ districts were 75% less likely to record clinical processes compared to the ‘2012 old’ districts (Table 4). Similarly, in 2012 rural districts were 68% less likely to record clinical processes compared to the Metro district. Auditing of specific diseases showed that compared to folders on diabetes, those on asthma were 48% less likely to have recorded clinical processes; folders on COPD were 3.47 times more likely to have recorded clinical processes (Table 4).

**TABLE 4:** Associations with positive responses in 2012 audit.

Variable	Univariable OR (95% CI)	Multivariable OR (95% CI)
New districts	0.28 (0.26–0.31)	0.25 (0.21–0.31)
Rural districts	0.35 (0.32–0.38)	0.32 (0.28–0.37)
**Disease category**		
Diabetes	1.00 (reference)	1.00 (reference)
Hypertension	1.51 (0.82–2.77)	1.57 (0.80–3.06)
Asthma	0.54 (0.26–1.10)	0.52 (0.24–1.10)
Epilepsy	1.19 (0.59–2.42)	1.21 (0.56–2.62)
COPD	3.07 (1.77–5.31)	3.47 (1.90–6.33)

OR, odds ratio; CI, confidence interval.

Total number of folders audited was 6450.

## Discussion

This article describes for the first time the results of a clinical governance initiative on the treatment of NCDs which has been ongoing in the Western Cape for four years. The findings demonstrate clear areas for service improvement, but also improving performance for facilities with a longer history of conducting this audit.

### Increasing coverage of the audit with time

More facilities than in previous years (129 in 2012 compared to 29 in 2009) and all six districts in the Western Cape were involved in the audit in 2012. This shows the willingness of facilities to participate in the audit and to improve services.

### Effect of audit on quality of care

The evaluation showed small to moderate improvements in clinical processes from 2009 to 2012. In cases where there was low baseline adherence to what is recommended and accepted as standard practice, audits have been shown to be very effective at improving care; where adherence was already high, there were smaller effects.^8,12^ This can partly explain the modest improvements seen over the years. It is evident from the facility audit that in the case of most of the processes initial adherence was already high in 2009, with some indicators (such as the availability of a functioning scale) being as high as 100%. Where there were no improvements, such as comparing 2011 to 2012, this could be attributed to the ‘newer’ health districts doing worse (‘2012 new’) than/the ‘older’ health districts (‘2012 old’).

Overall there were marked improvements in the ‘2012 old’ districts compared to the ‘2012 new’ districts on all eight clinical indicators for the folder review. Irrespective of the disease, ‘new’ districts had lower odds of recording clinical processes than the ‘old’ districts. The improvements seen are probably due to changes in practices at health districts where audits have been done for a longer period. This could be seen when the Metro district, the longest participating district in the province, was compared to the rural districts. If clinicians are given feedback about their practices, it will be expected that they will alter what they do if their clinical practice is found to be suboptimal and not according to the accepted standard and guidelines.^8^ It has been found that when this feedback was given intensively and regularly to all healthcare professionals and when it came from peers, the effects were greater.^8,23^ Such was the case in certain districts where the presence of good managerial support, dedicated champions and regular meetings where feedback was given frequently saw improvements in the audit processes (Van Vuuren U, Provincial Chronic Diseases programme coordinator, oral communication, 12 August 2013).

Furthermore, district NCD management forums were implemented to monitor improvement plans in the rural districts, and in the Metro district reorganisation of the PHC management meant that there was zero tolerance for the availability of minimum equipment. Patient care was also improved in the Metro through setting up appointment systems and strengthening dedicated days where patients were managed more intensively. It was also found that districts where auditing has been done for longer periods were likely to have permanent staff, and this contributed to stability in knowledge and skills gained and confidence in the clinical care given to patients (Van Vuuren U, oral communication, 12 August 2013).

### Room for improvement

Despite the improvements seen, a lot still needs to be done in improving the overall care given to patients with NCDs, especially in the case of asthma, which had 48% lower odds of clinical processes being recorded compared to diabetes. Also evident in the folder review is that the highest proportion achieved in 2012 was 64% for counselling for smokers with COPD, and only 28% for diabetic foot examination. This is not surprising, since previous audits and other studies done in the same context have shown shortfalls in the quality of care given to patients with NCDs, particularly those with hypertension and diabetes.^24,25^

Several barriers need to be overcome in order to improve the overall quality of care provided to patients with NCDs. Apart from health system issues, one of the major barriers to the successful translation of evidence into locally accepted policies lies in leaders and managers being ineffective and unaccountable.^26^ Mash et al.^25^ make a number of recommendations to improve chronic disease care, including building chronic care teams, involving the community, skills training for healthcare professionals and providing leadership for the chronic disease management teams.

### Integration with clinical governance for care of patients with other conditions

There are more and more patients suffering from both NCDs as well as communicable diseases. This is more so in South Africa with its quadruple burden of disease.^3^ Although this study analysed audit data for NCDs, audits can be used to improve care in patients with communicable diseases such as HIV and/or AIDS and TB.^27^

In moving forward it may be helpful to develop an integrated audit tool that not only looks at five NCDs (diabetes, hypertension, asthma, COPD and epilepsy), as was done here, but also looks at mental health as well as the communicable diseases for a more inclusive and comprehensive monitoring approach. This will need cross-programme collaboration and strengthening of partnerships with policy makers, healthcare professionals, public health researchers and funding organisations.^28,29^

### Limitations

The main limitation in this study was the fact that the audit was done internally by senior health professionals within each facility. This essentially led to the possibility of reporting bias. Furthermore, there was no internal or external validation. In 2011, 24 clinics were not included in the analysis; this was because they were unable to collect 10 folders for each chronic condition due to their small size. Data to differentiate clinics participating in the audit for the first time from those who had audited previously was only available in 2012. The associations between clinics with and without a history of having conducted the audit previously could be confounded by unmeasured factors such as clinic size and stability, which contributed both to their earlier participation in the audit process as well as better clinical care and management. Finally, an audit of this nature is not the best way to assess long-term clinical outcomes because of the sample size. A different research methodology will be required for assessing clinical outcomes such as strokes and amputations.

## Conclusion

Audits are done to create awareness about standards of care and for facilities to use their own data to identify areas where quality of care can be improved. Due to the efficiency of audits and quality of the data collected, audits are preferred over routinely collected data. The findings from this study have shown that audits done over time can significantly improve clinical processes in health districts. Even when the improvement is small, it may still be useful based on the context in which it was done.

However, beyond audits much still needs to be done to improve chronic disease care. Emphasis should be placed on facilities to set up multidisciplinary chronic care teams which will take responsibility for improving the chronic care that they provide. Community participation in the form of Community Health Committees should be part of healthcare service planning. Also, a patient experience questionnaire could be included in future audits to explore patient satisfaction with services.

Given the value of audits in improving care processes over the long term, it is recommended that the audit be extended to all PHC facilities in the Western Cape, and expanded to audit care processes across all chronic diseases. This will require political backing and dedication from health programmes within the Department of Health and from service providers as implementers.

It is anticipated that the improvements seen will translate into improved patient outcomes in the future.

## Cometing interests

The authors declare that they have no financial or personal relationship(s) that may have inappropriately influenced them in writing this article.
